# Phenotypic Subtyping and Re-analyses of Existing Transcriptomic Data from Autistic Probands in Simplex Families Reveal Differentially Expressed and ASD Trait-Associated Genes

**DOI:** 10.3389/fneur.2020.578972

**Published:** 2020-11-12

**Authors:** Valerie W. Hu, Chongfeng Bi

**Affiliations:** Department of Biochemistry and Molecular Medicine, The George Washington University School of Medicine and Health Sciences, Washington, DC, United States

**Keywords:** ASD subgroups, transcriptomic analysis, simplex families, ASD trait-associated genes, comparison with multiplex population

## Abstract

Autism spectrum disorder (ASD) describes a collection of neurodevelopmental disorders characterized by core symptoms that include social communication deficits and repetitive, stereotyped behaviors often coupled with restricted interests. Primary challenges to understanding and treating ASD are the genetic and phenotypic heterogeneity of cases that complicates all omics analyses as well as a lack of information on relationships among genes, pathways, and autistic traits. In this study, we re-analyze existing transcriptomic data from simplex families by subtyping individuals with ASD according to multivariate cluster analyses of clinical ADI-R scores that encompass a broad range of behavioral symptoms. We also correlate multiple ASD traits, such as deficits in verbal and non-verbal communication, play and social skills, ritualistic behaviors, and savant skills, with expression profiles using Weighted Gene Correlation Network Analyses (WGCNA). Our results show that subtyping greatly enhances the ability to identify differentially expressed genes involved in specific canonical pathways and biological functions associated with ASD within each phenotypic subgroup. Moreover, using WGCNA, we identify gene modules that correlate significantly with specific ASD traits. Network prediction analyses of the genes in these modules reveal canonical pathways as well as neurological functions and disorders relevant to the pathobiology of ASD. Finally, we compare the WGCNA-derived data on autistic traits in simplex families with analogous data from multiplex families using transcriptomic data from our previous studies. The comparison reveals overlapping trait-associated pathways as well as upstream regulators of the module-associated genes that may serve as useful targets for a precision medicine approach to ASD.

## Introduction

Autism spectrum disorder (ASD) is a neurodevelopmental disorder in which symptoms typically appear within the first 3 years of life. According to the Diagnostic and Statistical Manual of Mental Disorders-5th Edition (DSM-5), a diagnostic guide created by the American Psychiatric Association for mental disorders (1), individuals with ASD are affected in two core domains: social communication and repetitive, stereotyped behaviors often with restricted interests. DSM-5 differs from the previous DSM-4 diagnostic guide in that a third core domain (i.e., language development and pragmatics) is now integrated into social communication. Moreover, individuals who were previously classified into one of several related conditions, including Autistic Disorder, Asperger's Syndrome, and Pervasive Developmental Disorder-Not Otherwise Specified (PDD-NOS) are now combined under “Autism Spectrum Disorder.” Nonetheless, individuals with ASD manifest a wide variety of symptoms within the core domains, with highly variable severity. Thus, even though the clinical definition for ASD has been condensed, the genetic and phenotypic heterogeneity of affected individuals still complicates omics studies of ASD. In addition, there are no clear relationships between gene expression profiles and autistic traits in ASD individuals.

Recently, researchers have explored reducing clinical heterogeneity by selecting individuals with specific ASD traits based on severity scores from the Autism Diagnosis Interview-Revised (ADI-R) diagnostic instrument (2–5). For research purposes, the ADI-R diagnostic questionnaire is considered the gold standard behavioral test for ASD (6), whereas the DSM-5 is used for screening in clinical practice. The ADI-R, based on the DSM-4 guidelines, focuses on behavioral assessment in three main areas: reciprocal social interaction, communication and language, and repetitive stereotyped behaviors, including restricted interests. Previous studies in our laboratory identified four phenotypic groups in multiplex families (having more than one affected child) by multivariate cluster analyses of 123 severity scores on 63 items from the ADI-R (4). In addition, we validated biological differences among these subgroups by transcriptomic analyses of three of the subgroups in comparison to a control group (7). Moreover, class prediction analyses of differentially expressed genes (DEGs) from these analyses identified a limited number of DEGs that could differentiate cases from controls, thus exhibiting potential for use as biomarkers (8).

For genetic and quantitative trait association analyses, we further divided the three core domains probed by the ADI-R (social, language, and repetitive behaviors) into subcategories (including spoken language, non-verbal communication, play skills, social interactions, and insistence on sameness) which led to the identification of both subtype- and trait-associated genetic variants (9, 10). Savant skills, which are present in roughly 10% of individuals with ASD, is also a trait of interest and were present at higher frequency in one of the subgroups used for gene expression profiling (7).

Despite the demonstrated association of genetic variants and autistic traits, there is still relatively little understanding of the relationship between gene expression and ASD traits. In this regard, Weighted Gene Co-expression Network Analysis (WGCNA) is a software tool designed to explore the relationships between gene expression profiles and external information, such as case-control status or a specific condition (11). It has been applied recently in research on Alzheimer's disease (12), bipolar disorder (13), pancreatic cancer (14), and renal cell carcinoma (15) to identify gene networks dysregulated within the respective diseases. Co-expression analysis has also been implemented in previous research studies on ASD (16–21), but there has yet to be WGCNA analyses focused on autistic traits.

The primary goals of this study are to: (1) determine whether subtyping of the ASD probands from simplex families (having only one affected child) by cluster analyses of scores from the ADI-R improves the ability to determine gene expression (i.e., biological) differences between subtyped cases and controls over unsegregated cases and controls, (2) use WGCNA to investigate the association of gene networks with various traits of ASD, and (3) compare the gene expression modules and pathways associated with selected autistic traits in simplex families vs. those in multiplex families to identify both differences and similarities.

## Materials and Methods

**Figure 1:**
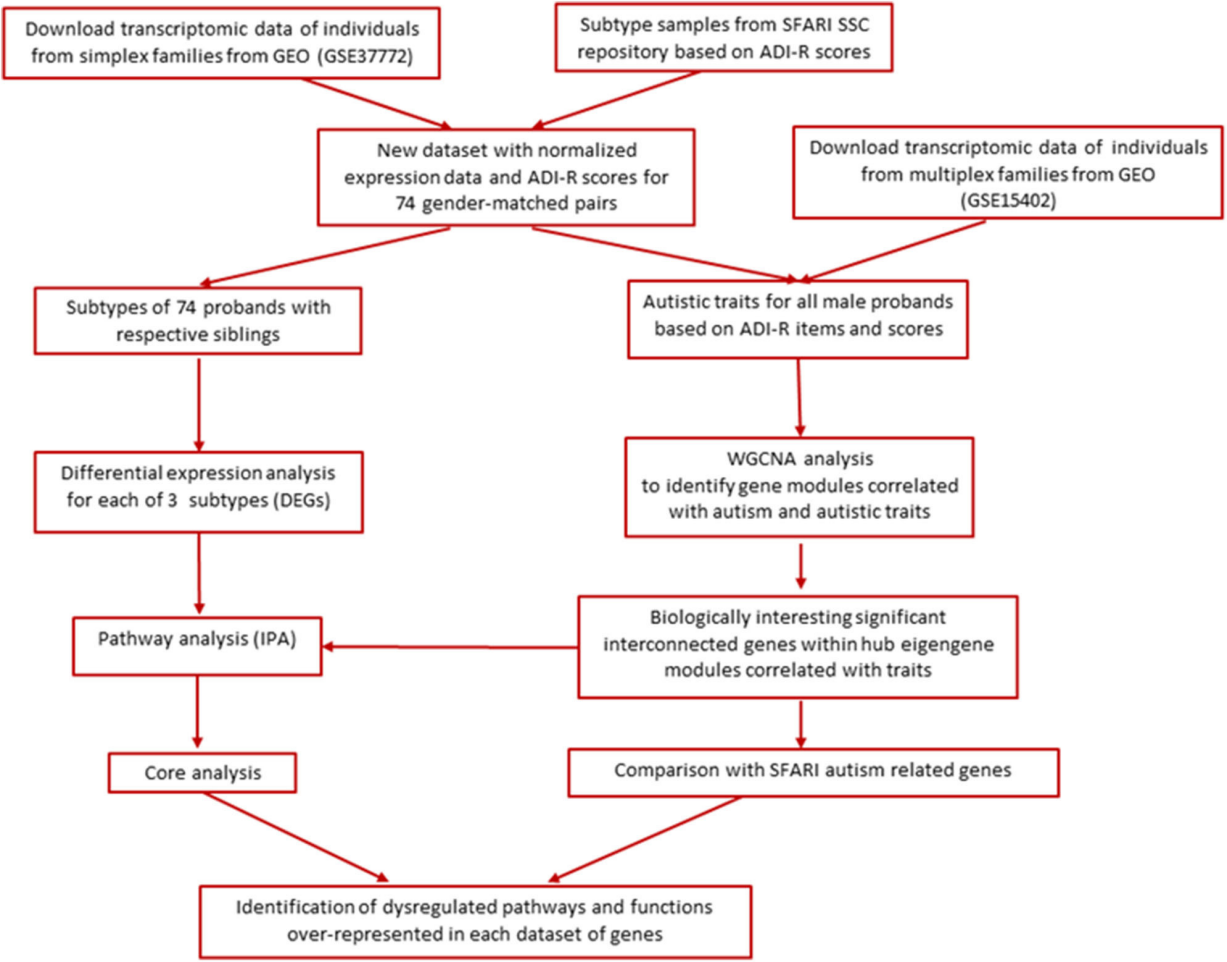
Overall analytical workflow.

The main goal of this study was to re-analyze existing transcriptomic data on a group of autistic probands and their respective siblings from the Simons Simplex Collection (SSC) in order to determine whether subtyping of ASD probands by multivariate cluster analysis of ADI-R item severity scores can facilitate detection of gene expression differences between cases from phenotypic subgroups and their non-autistic siblings. In addition, we sought to interrogate and compare relationships between gene networks and autistic traits in cases from both simplex and multiplex families. Figure 1 shows the overall analytical workflow for this study.

### Phenotypic Subtyping for Simplex Families

**Figure 2:**
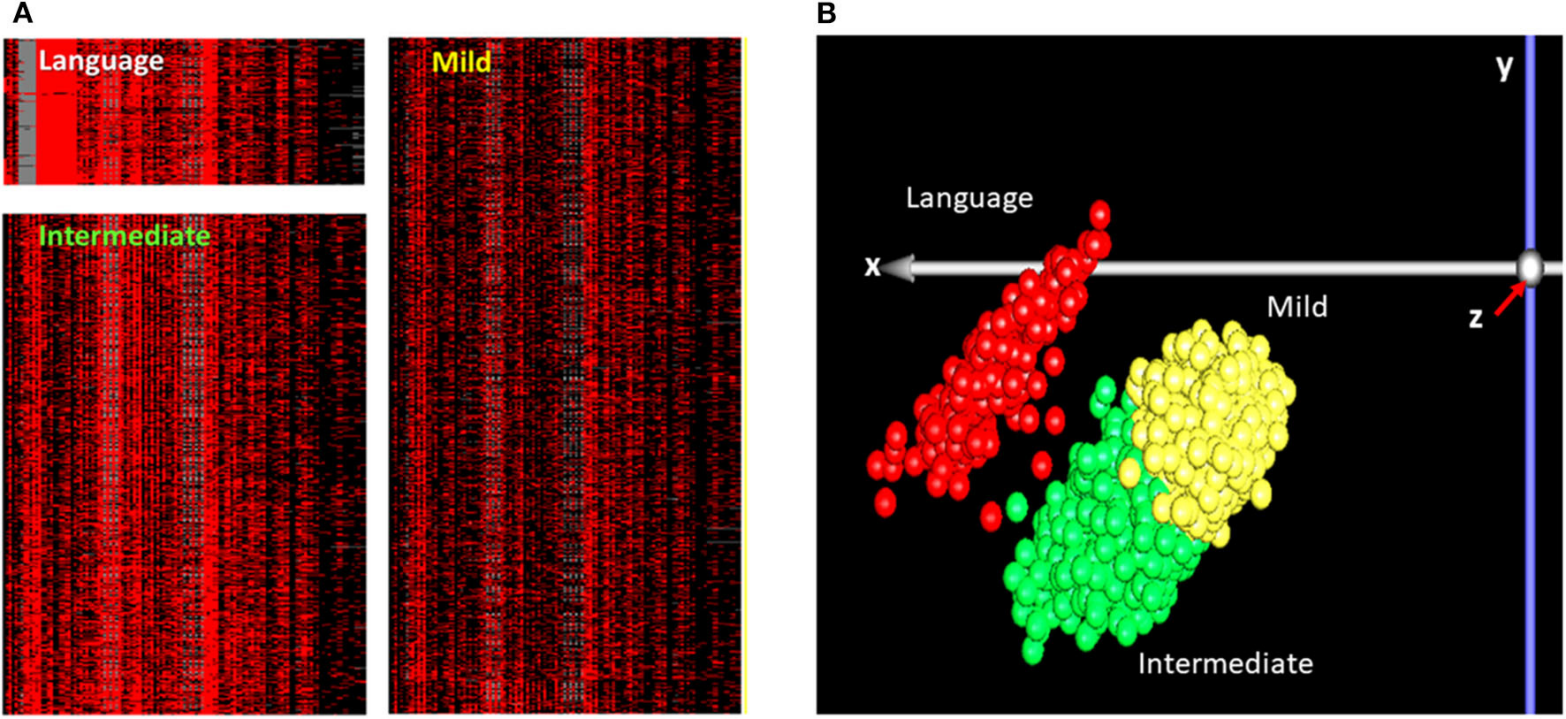
Phenotypic subgroups of individuals with ASD which were identified by: **(A)** K-means cluster (KMC) analysis of 123 scores on 63 ADI-R items for 1,900 individuals with ASD, with K = 3; **(B)** Graphical representation of a principal component analysis (PCA) of the ADI-R scores represented in panel **(A)** for 1,900 ASD cases from the SSC. For the KMC analysis **(A)**, each row represents an individual with ASD and each column represents the respective individual's score on a specific ADI-R item. Severity of ADI-R item scores are indicated by the intensity of red in the heatmaps, with bright red indicating a score of 3, lighter shades of red indicating scores of 2 and 1, and black representing a score of 0, which is equivalent to “normal.” The bright red block of columns in the “Language” subgroup corresponds to items related to deficits in spoken language on the ADI-R diagnostic instrument. Based on the KMC analysis, the subgroups were labeled as “Language” for the severely language-impaired subgroup, “Mild” for the subgroup with the lowest severity profile across the 123 ADI-R scores, and “Intermediate” for the subgroup exhibiting an intermediate severity profile between the Language-impaired and Mild subgroups. PCA was used to reduce the dimensionality of the multivariate data (i.e., 123 scores per individual) in order to better visualize the clusters of individuals with similar severity profiles across all ADI-R items. The *x, y*, and *z* axes represent the first, second, and third principal components from the PCA analysis **(B)**. Each point on the graph represents an individual, with red representing individuals in the Language subgroup, green representing individuals in the Intermediate subgroup, and yellow representing individuals in the Mild subgroup.

Raw ADI-R scoresheets for 1,900 individuals with ASD (i.e., probands) were obtained from the SSC (New York, NY). As described previously for multiplex families (4), 123 scores on 63 ADI-R items for each proband were subjected to *K*-means cluster (KMC) analysis. This analysis showed an optimum separation of cases with *K* = 3, indicating three phenotypic subgroups. This was in contrast to the four phenotypic subgroups that optimally distinguished ASD cases in multiplex families (4). Unsupervised principal component analysis (PCA), a dimensionality reduction tool, was then used to more clearly visualize the distribution of cases from these three subgroups based on their respective ADI-R severity scores (Figure 2).

### Acquisition of Transcriptomic Data for Individuals With ASD and Their Siblings

Normalized gene expression data from a study by Luo et al. (22) on lymphoblastoid cell lines (LCL) from 412 individuals (combined cases and controls) included in the SSC were downloaded from the Gene Expression Omnibus (GEO) data repository using the GEOquery R package (accession number GSE37772). The expression data had been obtained using the Illumina Whole Human Genome Array Human REF-8 version 3.0 following the manufacturer's standard protocol (22). Among the set of individuals included in the study were 168 pairs of cases and controls (each pair from the same family), including 98 sibling pairs matched for sex. The sample identification numbers (IDs) of the probands in this group were cross-referenced against those for whom complete ADI-R scoresheet data were available for the subtyping analyses described above. This resulted in the identification of 74 pairs of sex-matched cases and sibling controls with both gene expression and ADI-R data (for cases only). The demographic information on these individuals is shown in Supplementary Table 1. Based on the cluster analyses of ADI-R scores, the probands (together with their respective unaffected siblings) were distributed into three phenotypic subgroups for transcriptomic analyses. These subgroups were identified as: Language (7 pairs), Intermediate (26 pairs), and Mild (41 pairs).

For WGCNA analyses of autistic traits in individuals with ASD from multiplex families, normalized gene expression data from a study by Hu et al. (7) were downloaded from GEO (accession number GSE15402). These data were obtained using custom-printed TIGR 40K human arrays containing 39,936 human cDNA probes, as previously described. ADI-R data for the individuals in this study were obtained from the Autism Genetics Research Exchange, with subtyping performed on adjusted ADI-R scores as previously described (4).

### Identification of Subgroup-Associated Differentially Expressed Genes (DEGs)

Differential gene expression analysis was performed using Multi-experiment Viewer (MeV) software for microarray analyses (23) which employed an 80% data filter, meaning that each gene included in the study had an expression value in at least 80% of the samples. *T*-tests with bootstrapping permutation, which randomly regrouped samples 1,000 times, were conducted on the normalized data from cases within each subgroup and their respective sibling controls to identify significant DEGs with a critical *p*-value set at ≤ 0.05. Principal component analysis (PCA) was used to visualize the separation of cases and controls based on the subgroup-associated DEGs. Based on the PCA results, we used a more stringent *p*-value of ≤ 0.03 to optimize the separation of Mild cases from their respective sibling controls.

### Classification of Clinical Autistic Traits

Another goal of this study was to investigate the correlation between gene expression profiles and autistic traits. The three core domains considered in the ADI-R diagnostic tool (communication and language, social interaction, and repetitive stereotyped behaviors were further parsed into six primary traits as shown in Supplementary Table 2, along with the ADI-R items corresponding to each trait. The cumulative severity score across the items comprising each trait was used as the specific trait score for each individual. The six traits included impairment of verbal communication (Verbal), non-verbal communication (Non-verbal), social interaction (Social), play skills (Play), insistence on sameness and rituals (Sameness), and presence of savant skills (Savant).

### Weighted Gene Co-expression Network Analysis (WGCNA)

WGCNA is an R-package primarily designed for co-expression analysis of transcriptomic data (11). In this study, WGCNA analyses were performed on normalized expression data from all detectable genes to identify correlated gene networks associated with each of the autistic traits in both simplex and multiplex families. The threshold of signed *R*^2^ was set to 0.85 prior to network construction, as this value is used to calculate the unsigned co-expression topology overlap necessary for constructing the network. Gene clusters, called modules, were then merged if the correlation values for the eigengenes were above 0.8. Hub modules were selected based on significant correlation (Pearson's correlation coefficient, *p* ≤ 0.05) with external information (i.e., autistic trait severity scores). For WGCNA analyses of autistic traits in simplex families, normalized gene expression data for 24,526 probes (corresponding to 18,415 genes) from 63 male probands were used to identify gene networks that associated with the six autistic traits. Similarly, WGCNA analysis of autistic traits in multiplex families utilized normalized gene expression data for 28,592 probes (corresponding to 11,129 genes) from 81 male individuals with ASD. This transcriptomic data was derived from the study of 7).

### Pathway and Functional Analyses of DEGs and Module Genes From WGCNA

Ingenuity Pathway Analysis (IPA) software (Qiagen, Germantown, MD) was used for network prediction analyses to identify canonical pathways, biological functions, and diseases enriched among DEGs from our subtype-dependent transcriptomic analyses as well as those over-represented among genes in significant trait-associated gene modules from WGCNA analyses. The Fisher Exact Test, as implemented by IPA software, was used to determine the significance of enrichment with respect to a given pathway, function, or disease using IPA Knowledgebase as a reference gene set. IPA was also used for comparison analysis of pathways and upstream regulators associated with DEGs from simplex and multiplex samples.

### Hypergeometric Distribution Analyses for ASD Gene Enrichment Among Module Genes

Trait-associated module genes were compared to a list of 910 known autism risk genes in the SFARI Gene database (24). Venny 2.1.0, an online software package for creating Venn diagrams (25), was used to identify overlapping genes between the SFARI gene set and the trait genes https://bioinfogp.cnb.csic.es/tools/venny/. The CASIO Keisan Online Calculator http://keisan.casio.com/exec/system/1180573201 was then used to determine hypergeometric distribution probabilities for over-representation of module genes within the SFARI dataset with significance determined by an upper cumulative *q* ≤ 0.05.

## Results

### Differential Gene Expression Analysis of Each Subtype in Simplex Families

**Figure 3:**
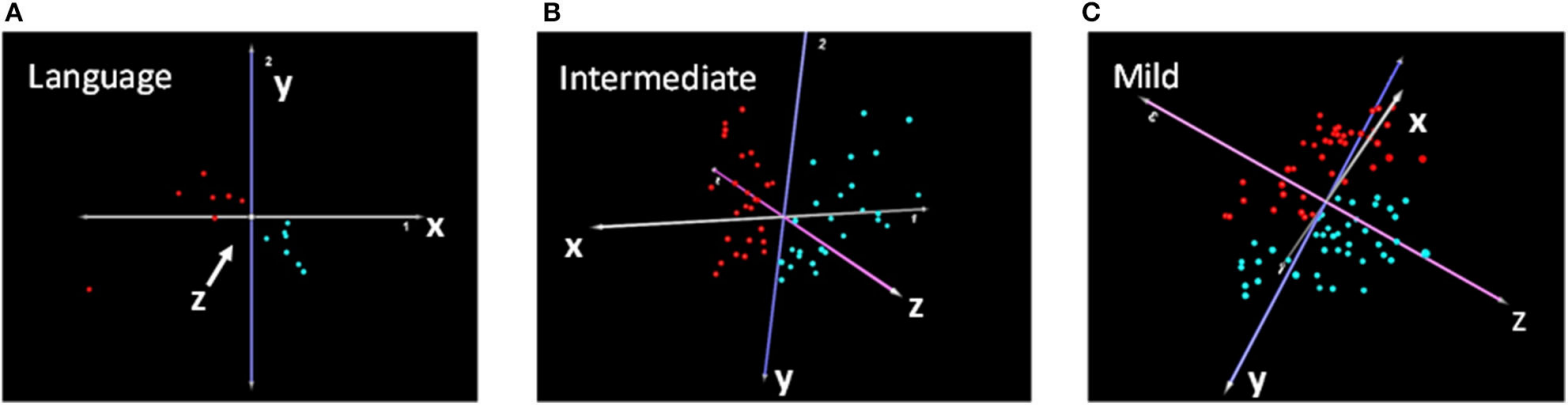
Principal component analyses showing separation of cases (red) and controls (turquoise) based on DEGs from transcriptomic analyses of the **(A)** Language, **(B)** Intermediate, and **(C)** Mild cases and their respective sibling controls.

Differential gene expression analysis of each subtype shown in Figure 2 resulted in a total of 774, 384, and 274 differentially expressed transcripts corresponding to 765, 377, and 270 DEGs for the Language, Intermediate, and Mild subgroups, respectively (Supplementary Tables 3–5). PCA analyses based on these subtype-associated DEGs showed almost complete separation of cases from controls within each phenotypic subgroup, as illustrated in Figure 3. In addition, a separate gene expression analysis of all cases (combined subgroups) and controls was performed to assess the result of heterogeneity reduction in identifying DEGs and associated biological processes (Supplementary Table 6).

### Pathway Analysis of DEGs From Each Subtype in Simplex Families

IPA was used to identify over-represented canonical pathways, diseases, and neurological functions among the DEGs from each of the three ASD subgroups. Table 1 shows a comparison of autism-relevant canonical pathways significantly enriched among DEGs from the three subgroups. As shown, there are a much greater number of significantly over-represented pathways relevant to ASD in the Language subgroup in comparison to those over-represented in the Intermediate and Mild subgroups. Notably, all of the pathways shown are also enriched among genes from the SFARI Gene database (data not shown). Interestingly, there were no significant autism-associated pathways enriched among DEGs obtained when all 74 cases were combined for expression analysis as shown in Supplementary Table 7, which provides a complete list of significant pathways for both subgroup and combined group analyses.

**Table 1:** Comparison of significantly over-represented ASD-relevant canonical pathways in three phenotypic subgroups of ASD (Language, Intermediate, and Mild).

**Ingenuity canonical pathways**	**Language**	**Intermediate**	**Mild**
**(implicated in ASD)**	**–log(*****p***-value)^*^		
Axonal Guidance Signaling	3.61		1.44
Protein Kinase A Signaling	3.44		
Actin Cytoskeleton Signaling	3.33		
CDK5 Signaling	3.22		
cAMP-mediated signaling	2.69	1.67	
Androgen Signaling	2.46		
Synaptogenesis Signaling Pathway	2.03		2.14
Melatonin Signaling	2.02		
VDR/RXR Activation	2.00		
Ephrin Receptor Signaling	1.99		
Neuregulin Signaling	1.93	1.78	2.36
GABA Receptor Signaling	1.93		
Gap Junction Signaling	1.90		
CREB Signaling in Neurons	1.84		
Synaptic Long Term Depression	1.74		
PI3K/AKT Signaling	1.66		
Synaptic Long Term Potentiation	1.62		
Estrogen Receptor Signaling	1.58		
Neurotrophin/TRK Signaling	1.44		
ERK/MAPK Signaling	1.41		
Netrin Signaling		1.71	
PTEN Signaling		1.39	

* *Negative logarithm of the Fisher Exact p-value indicating the probability that the indicated pathway is not over-represented among the genes in the respective datasets. A –log(p-value) of 1.3 is equivalent to a p-value of 0.05. Only significant values are shown*.

Table 2 shows an IPA-generated comparison of over-represented neurological diseases and functions that are both shared and unique among the ASD subtypes and the combined case group. Not surprisingly, the Language subgroup is associated with more neurological functions than either of the other two subgroups or the combined group. This finding is consistent with the phenotypic data which suggests that the Language subgroup is the most severely affected according to ADI-R severity scores (see Figure 2). Cognitive impairment also occurs exclusively within the confines of the Language subtype. Interestingly, sensory disorders and epilepsy, which are cormorbidities frequently associated with ASD (26, 27), are enriched within DEGs in the Intermediate and Mild subgroups, respectively. None of these subgroup-associated comorbid disorders were identified when all cases were combined.

**Table 2:** Comparison of significantly over-represented neurological functions and diseases in three phenotypic subgroups of ASD as well as in the combined case group.

**Neurological diseases and functions**	**Language**	**Intermediate**	**Mild**	**Combined**
	**–log(*****p***-value)^*^			
Morphology of nervous system	6.49	4.52		3.09
Organization of cytoskeleton	6.03			
Motor dysfunction or movement disorder	5.82			
Neurological signs	5.60			
Neurotransmission	5.59	3.68		2.37
Movement Disorders	5.58			
Schizophrenia spectrum disorder	5.35		3.16	
Severe psychological disorder	5.11			
Abnormal morphology of nervous system	4.90	4.11		2.32
Cognition	4.89	3.87		2.99
Synaptic transmission	4.73	3.22		
Memory	4.36	2.80		
Potentiation of synapse	4.12			
Morphology of brain	3.93	3.27		
Learning	3.90	2.92		2.78
Neuronal cell death	3.87			
Cognitive impairment	3.86			
Long-term potentiation	3.83			
Development of neurons	3.77		2.54	
Organization of actin cytoskeleton	3.71			
Growth of neurites	3.64			
Neuritogenesis	3.38			
Outgrowth of neurites	3.24			
Secretion of neurotransmitter	3.17		3.40	
Morphology of dendritic spines	3.09			
Polarization of neurites	3.04			
Complete agenesis of corpus callosum		3.67		
Extension of dendrites		3.33		
Sensory disorders		3.25		
Epilepsy or neurodevelopmental disorder			4.58	2.90
Seizure disorder			3.41	
Epilepsy			3.35	
Polarization of neuroglia			3.18	
Complexity of dendritic trees				4.10
Migration of neurons				3.46
Size of neurons				3.40
Size of dendritic trees				3.38
Guidance of axons				3.19
Sensory system development				3.06
Emotional behavior				3.04

* *Negative logarithm of the Fisher Exact p-value which indicates the probability that the indicated disease or function is not over-represented in the respective dataset of genes. A –log(p-value) cutoff of 3 which is equivalent to a p-value of 0.001 was used to select diseases and functions from each group shown here*.

### WGCNA Analyses of Autistic Traits in Simplex Families

**Figure 4:**
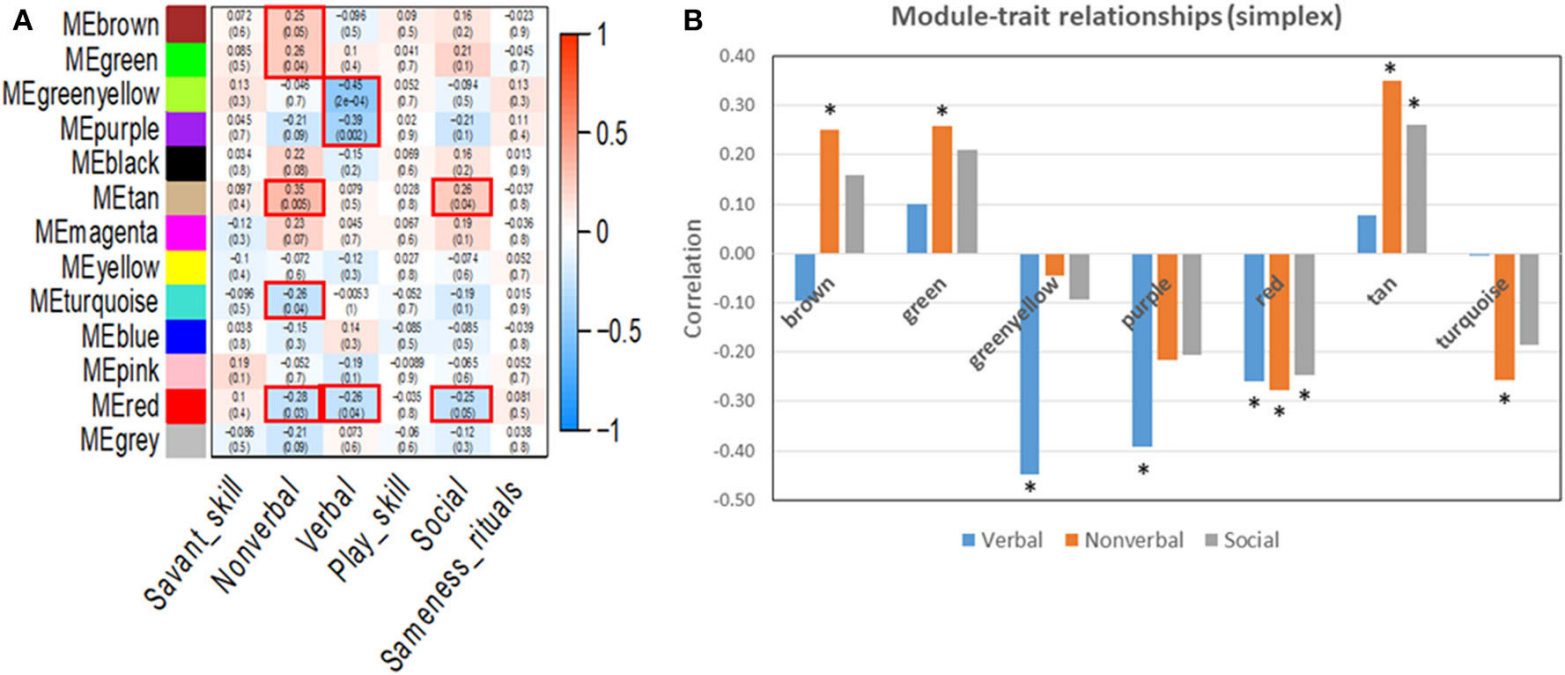
**(A)** Module-trait relationships resulting from WGCNA analyses of autistic traits in the simplex population. Significant modules with *p* ≤ 0.05 are outlined in red. **(B)** Correlation values for the trait-associated gene modules that are significantly correlated with at least one autistic trait. ^*^p ≤ 0.05. Each colored module represents a network of genes with correlated expression among its members. Six autistic traits were evaluated for correlated gene expression networks. Three traits (verbal, non-verbal, and social) showed significant association with at least one module.

WGCNA was used to identify gene clusters (modules) correlated with clinical autistic traits in 63 male probands using their respective cumulative ADI-R trait scores and normalized expression data for all detectable genes. Inasmuch as ASD is a neurodevelopmental disorder predominantly affecting males (28), we decided to focus on male probands to avoid confounding effects related to sex. Module-trait relationships determined by WGCNA revealed that only the verbal, non-verbal, and social interaction traits were significantly correlated with specific gene modules (Figure 4). Pathway analyses were then conducted on all genes within the significant modules for each of these traits as described below. These included the greenyellow, purple, and red modules for the verbal trait, brown, green, red, tan, and turquoise modules for the non-verbal trait, and the red and tan modules for the social trait.

### Network Prediction Analyses of Genes Associated With Autistic Traits in the Simplex Population

**Figure 5:**
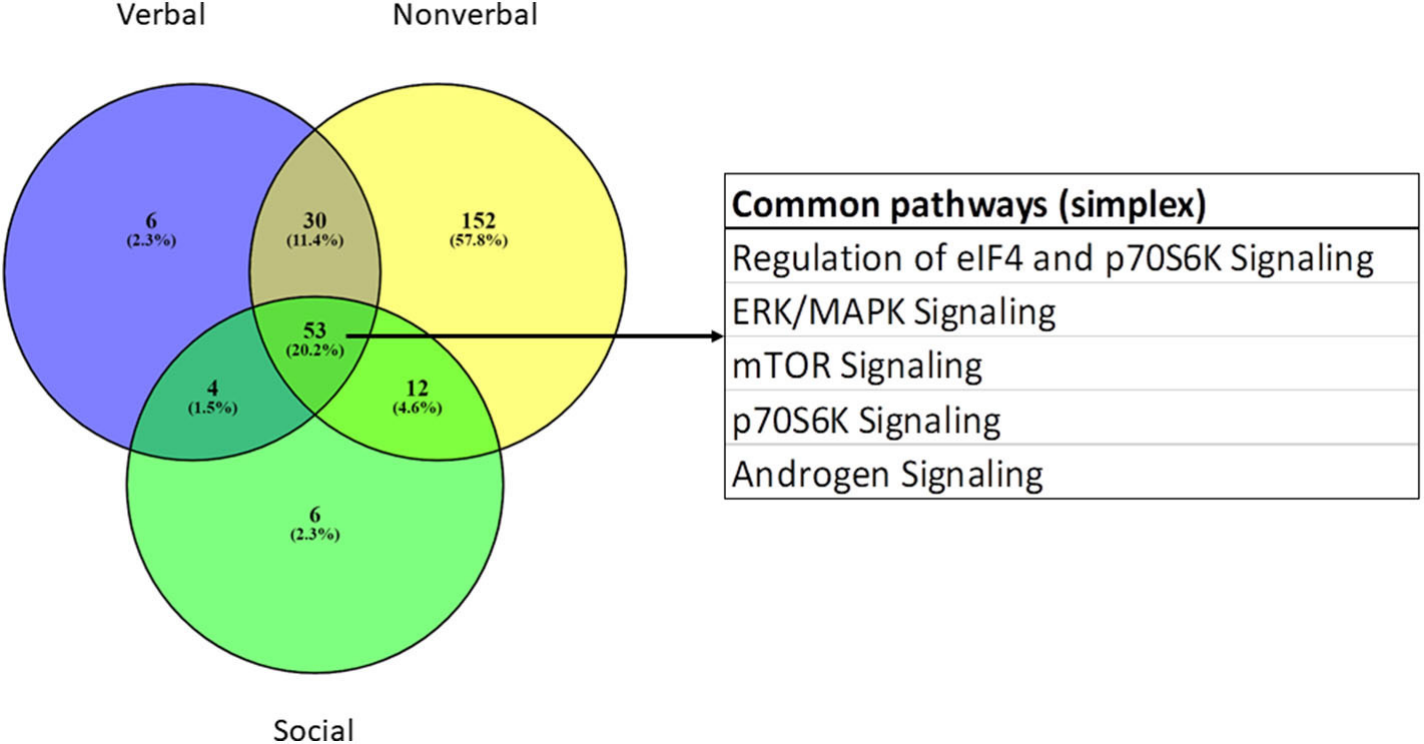
Venn diagram showing overlap of all over-represented canonical pathways associated with genes correlated with verbal, non-verbal, and social traits in the simplex population.

IPA was used to identify canonical pathways that were enriched among the module genes collectively contributing to verbal, non-verbal, and social deficits in individuals with ASD from the simplex population. The complete sets of significantly over-represented pathways for each trait are presented in Supplementary Table 8 together with the pathways associated with the combined ASD traits, using genes from all seven significant modules in an IPA core analysis. Table 3 summarizes the ASD-relevant canonical pathways among the top 25 over-represented pathways associated with verbal, non-verbal and social traits, while Figure 5 shows the overlap among all significant pathways associated with each trait. As shown, there are both unique and overlapping trait-associated pathways, with the non-verbal trait exhibiting the largest number of pathways previously implicated in ASD. Aside from the pathways shared among the three traits, the non-verbal trait is also uniquely associated with several neurological functions including reelin, NGF, neurotrophin, and synaptogenesis signaling in addition to estrogen receptor and NFkB signaling. Interestingly, mitochondrial dysfunction, which affects some individuals with ASD (29–32), was uniquely associated with deficits in social interaction. With respect to neurological diseases, only modules correlated with the non-verbal trait are enriched with genes that are significantly associated with mental retardation (*p* = 9.25E-13; 189 genes) and cognitive impairment (*p* = 1.50E-09; 265 genes), which are comorbidities frequently seen in ASD (27, 33). In addition, non-verbal communication is the only trait correlated with module genes that are explicitly associated with autism spectrum disorder or intellectual disability (*p* = 1.18E-13; 237 genes), suggesting that deficits in non-verbal communication are major contributors to the overall phenotype of ASD in the simplex population.

**Table 3:** Significantly over-represented ASD-relevant pathways among the top 25 associated with the verbal, non-verbal, and social traits in the simplex population.

**Ingenuity canonical pathways**	–log(*p*-value)^*^
**Verbal communication**	
Regulation of eIF4 and p70S6K Signaling	5.07
ERK/MAPK Signaling	4.86
EIF2 Signaling	4.86
mTOR Signaling	3.82
**Non-verbal communication**	
Regulation of eIF4 and p70S6K Signaling	8.35
Protein Ubiquitination Pathway	7.5
mTOR Signaling	7.48
Ephrin Receptor Signaling	7.21
Estrogen Receptor Signaling	6.8
EIF2 Signaling	6.51
Reelin Signaling in Neurons	6.25
p70S6K Signaling	6.12
NGF Signaling	6.09
Synaptogenesis Signaling Pathway	5.94
ERK/MAPK Signaling	5.92
**Social interaction**	
ERK/MAPK Signaling	3.34
ATM Signaling	3.06
Androgen Signaling	2.95

* *Negative logarithm of the Fisher Exact p-value which represents the probability that the pathway is not over-represented among the respective dataset of genes, where a value of 1.3 is equivalent to a p-value of 0.05*.

### WGCNA Analysis of Autistic Traits in Multiplex Families

**Figure 6:**
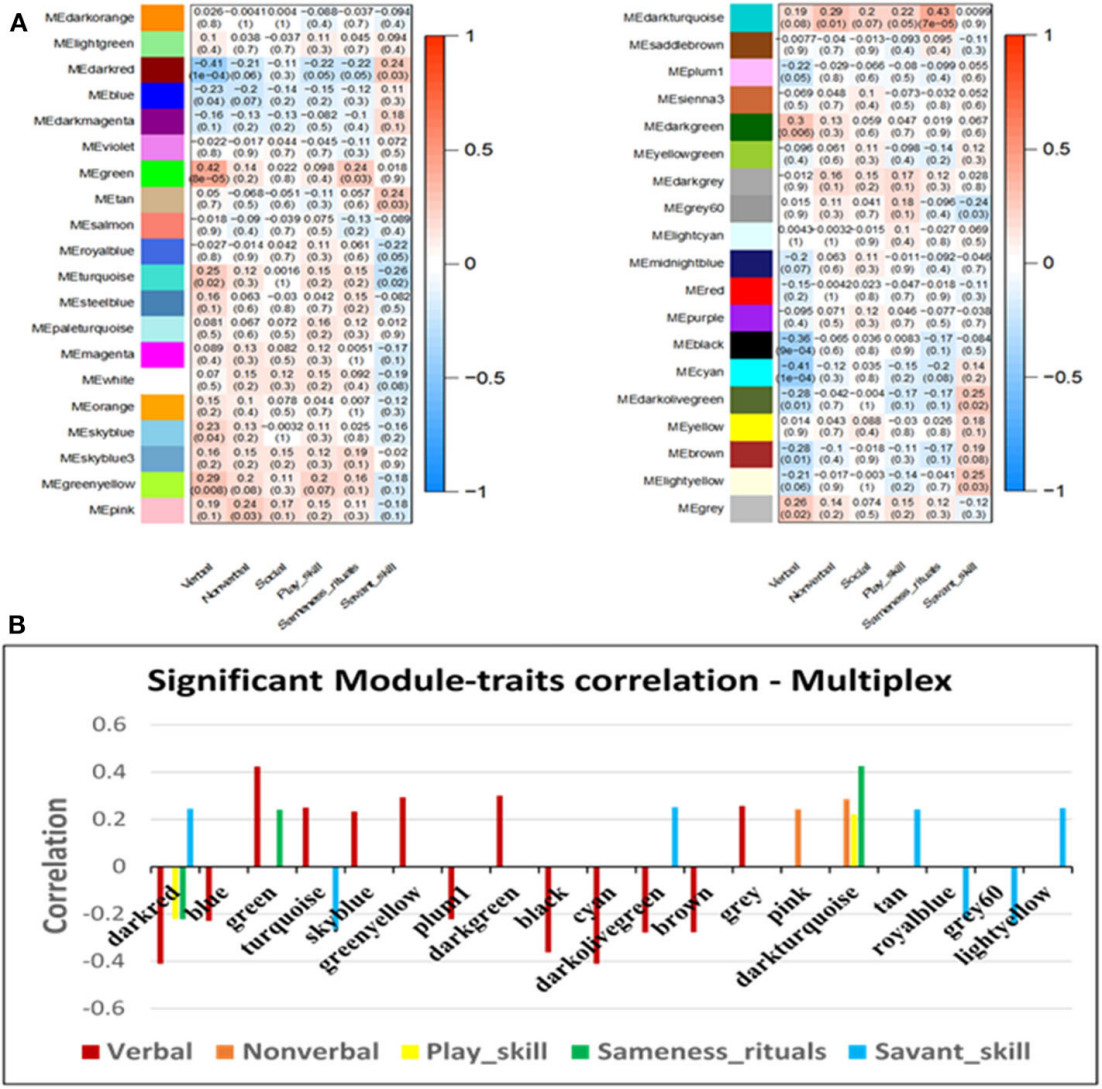
**(A)** Module-trait relationships resulting from WGCNA analyses of autistic traits in the multiplex population. **(B)** Correlation values for the trait-associated gene modules that are significantly correlated with at least one autistic trait, with *p* ≤ 0.05. Each colored module represents a network of genes with correlated expression among its members. Six autistic traits were evaluated for correlated gene expression networks. Five autistic traits (verbal, non-verbal, play, insistence on sameness or rituals, and savant) showed significant association with at least one module.

WGCNA was also used to identify gene modules that correlated with selected autistic traits in males from multiplex families that were included in our previous gene expression study of ASD subtypes (7) for the purpose of comparison with the data from simplex families in this study. Figure 6A shows that, in contrast to the results for probands from simplex families, more modules (18 in all) are highly correlated with five autistic traits. The significant modules and their correlation with each of the five traits (verbal, non-verbal, play, insistence on sameness and rituals, and savant skills) are highlighted in the barplot (Figure 6B). Interestingly, modules correlating with savant skills show inverse correlation with respect to other autistic traits sharing the same modules. By contrast, no modules were correlated with savant skills in the simplex population.

### Network Prediction Analyses of Genes Associated With Autistic Traits in the Multiplex Population

**Figure 7:**
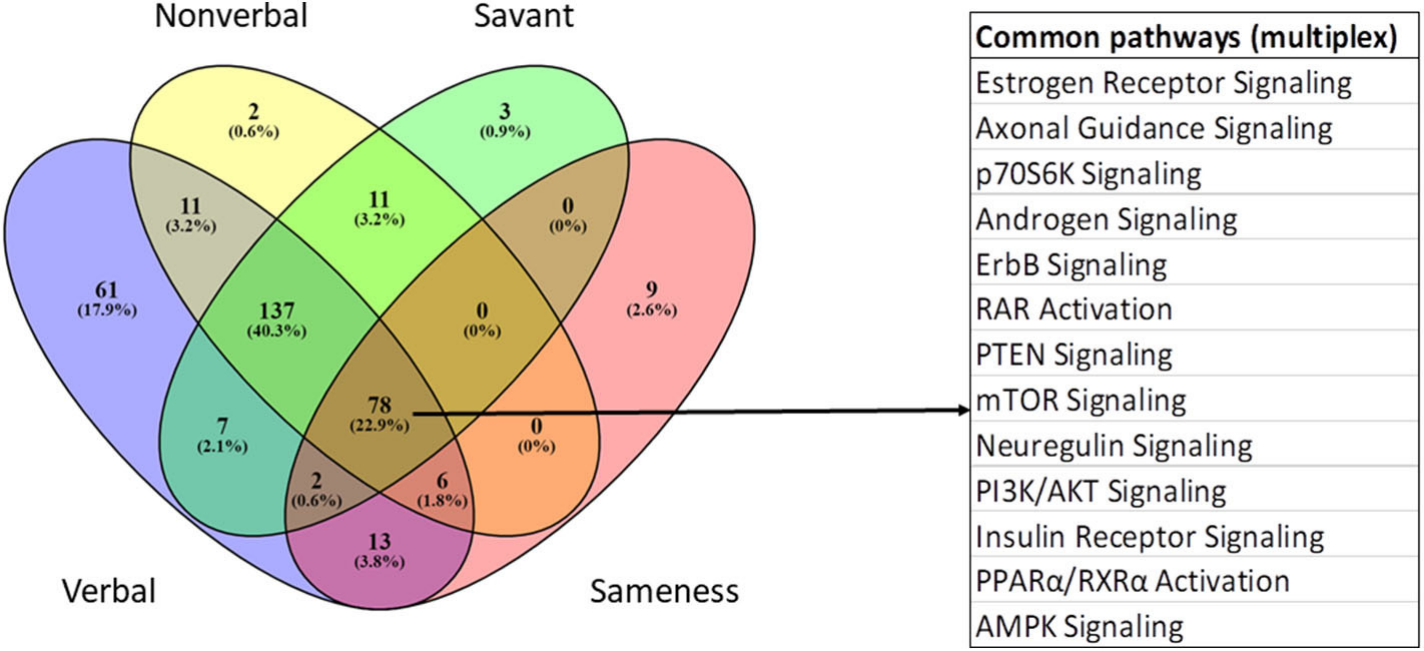
Venn diagram showing overlap of all over-represented canonical pathways associated with genes correlated with verbal, non-verbal, savant, and insistence on sameness traits in the multiplex population.

IPA was used to identify pathways and functions over-represented among module genes significantly associated with five ASD traits in the multiplex population. The gray module was not included in the pathway analysis because it represents the default module comprised of genes not correlated in any other module. The complete lists of significant trait-associated pathways are provided in Supplementary Table 9. Table 4 summarizes the ASD-relevant pathways among the top 25 over-represented pathways associated with four autistic traits (verbal, non-verbal, insistence on sameness, and savant skills), while Figure 7 shows the overlap among all significant pathways associated with the four traits. Although several metabolic pathways were significantly enriched for play skills, there were no neurological signaling pathways that are relevant to ASD. In contrast to the results obtained with the simplex population, there are more ASD-related pathways associated with the verbal than with the non-verbal traits. Estrogen receptor signaling and the protein ubiquitination pathway are among the most significantly over-represented pathways associated with three of the five traits (verbal, non-verbal, and savant), with –log(*p*-values) for pathway enrichment ranging from 7.9 to 12.5 and from 8.6 to 11.3, respectively. Mitochondrial dysfunction and oxidative phosphorylation are also significantly over-represented among genes associated with the non-verbal and savant traits. As for the simplex samples, there are both unique and overlapping ASD-associated pathways among the four traits represented in Figure 7. There is also overlap with the shared trait-associated pathways from the simplex analyses (Figure 5), but with additional pathways, including estrogen receptor signaling, axon guidance, PTEN, PI3K/AKT, and neuregulin signaling, shared among all four traits in the multiplex population.

**Table 4:** Significantly over-represented ASD-relevant pathways among the top 25 associated with four autistic traits in the multiplex population.

**Ingenuity canonical pathways**	–log(*p*-value)^*^
**Verbal communication**	
Estrogen Receptor Signaling	12.5
Axonal Guidance Signaling	11.1
Protein Kinase A Signaling	10.8
Synaptogenesis Signaling Pathway	9.92
p70S6K Signaling	9.81
Regulation of eIF4 and p70S6K Signaling	9.65
Androgen Signaling	9.17
Protein Ubiquitination Pathway	8.64
**Non-verbal communication**	
Protein Ubiquitination Pathway	10.7
Estrogen Receptor Signaling	10.4
Mitochondrial Dysfunction	8.06
Oxidative Phosphorylation	7.91
**Play skills**	
None specifically associated with ASD	
**Insistence on sameness (ritualistic behavior)**	
NF-κB Signaling	4.06
PTEN Signaling	2.31
Androgen Signaling	2.1
**Savant skills**	
Protein Ubiquitination Pathway	11.3
Mitochondrial Dysfunction	8.44
Oxidative Phosphorylation	8.39
Estrogen Receptor Signaling	7.9
Regulation of eIF4 and p70S6K Signaling	6.05
Androgen Signaling	5.97
ERK/MAPK Signaling	5.25
Protein Kinase A Signaling	5.16

* *Negative logarithm of the Fisher Exact p-value which represents the probability that the pathway is not over-represented among the respective dataset of genes, where a value of 1.3 is equivalent to a p-value of 0.05*.

With respect to neurological diseases, motor dysfunction and movement disorders are significantly associated with verbal (*p* = 5.7E-22), non-verbal (*p* = 3.69E-09), and savant (*p* = 8.36E-10) traits, while a related disorder, ataxic gait, is associated with play skills (*p* = 4.09E-04) and insistence on sameness or ritualistic behaviors (*p* = 5.79E-06). Genes for cognitive impairment are enriched for both verbal (*p* = 1.9E-15) and savant (*p* = 1.26E-08) traits, although the shared modules that are significant for these two traits show inverse correlation with respect to gene expression and cumulative trait score.

To better distinguish pathways differentiating verbal and savant skills, IPA was used to analyze genes in modules unique to each trait (i.e., not shared with any other trait). For the verbal trait, these 8 modules included the black, blue, brown, cyan, darkgreen, greenyellow, plum1, and skyblue. For the savant trait, these modules included grey60, lightyellow, royalblue, and tan. Table 5 shows the most significant ASD-relevant pathways among the top 50 associated with each set of trait genes. Interestingly, the pathways associated with the verbal trait are enriched in genes involved in neuronal processes (e.g., axon guidance, synaptogenesis, reelin, ephrin receptor, and neuregulin signaling), whereas the top pathways associated with the savant trait are related to NFkB, PI3K/AKT, ERK/MAPK, and downstream signaling pathways involved in inflammation, protein synthesis and cell growth. While both traits are associated with genes involved in motor dysfunction and movement disorders (*p* = 2.63E-11 for verbal; *p* = 7.30E-03 for savant), the savant skills are also associated with genes linked to severe mental retardation (*p* = 2.00E-03) and cognitive impairment (*p* = 4.89E-03), thus highlighting their relevance to intellectual ability and cognition.

**Table 5:** Significantly over-represented canonical pathways among genes in modules unique to verbal and savant traits.

**Ingenuity canonical pathways**	–log(*p*-value)^*^	**Ingenuity canonical pathways**	**–log(*p*-value)**
**(modules unique to verbal)**		**(modules unique to savant)**	
Axonal Guidance Signaling	11.1	NF-κB Signaling	4.49
Synaptogenesis Signaling Pathway	7.54	PI3K/AKT Signaling	2.68
Protein Kinase A Signaling	6.44	ERK/MAPK Signaling	2.40
Wnt/β-catenin Signaling	5.49	CREB Signaling in Neurons	2.14
p70S6K Signaling	5.42	PTEN Signaling	2.00
Reelin Signaling in Neurons	4.94	Protein Kinase A Signaling	1.98
Ephrin Receptor Signaling	4.91	Regulation of eIF4 and p70S6K Signaling	1.97
PTEN Signaling	4.67	p70S6K Signaling	1.88
EIF2 Signaling	4.19	Androgen Signaling	1.81
Neuregulin Signaling	4.14	mTOR Signaling	1.69

* *Negative logarithm of the Fisher Exact p-value which represents the probability that the pathway is not over-represented among the respective dataset of genes, where a value of 1.3 is equivalent to a p-value of 0.05*.

### Comparison of Genes, Biological Pathways, and Functions Associated With Autistic Traits in Simplex and Multiplex Populations and Their Relevance to ASD

While the WGCNA analyses of autistic traits clearly show differences in the gene networks associated with ASD in simplex and multiplex families, we also sought to find similarities as well as differences among all the trait-associated genes and biological networks. A direct comparison of all trait-associated genes from the simplex (4,649 genes) and multiplex (6,081 genes) individuals with ASD shows an overlap of 1,754 genes. Hypergeometric distribution analyses were then conducted to determine the over-representation of 910 autism risk genes (downloaded in May, 2020) from the SFARI Gene database within each set of trait-associated genes as well as the overlapping set of genes. These analyses show that trait genes from both simplex and multiplex populations as well as the overlapping set are significantly enriched in SFARI genes, with hypergeometric distribution upper cumulative *q*-values of 0.012 (simplex), 7.62E-06 (multiplex), and 0.044 (overlapping), thus confirming relevance of these genes to ASD.

IPA was then used to identify canonical pathways over-represented among the overlapping genes. Table 6 shows that the 1,754 shared trait genes are over-represented in pathways that reflect many of the top pathways enriched among module genes correlated with all ASD traits in simplex and multiplex populations (Table 7). Despite the fact that there are hundreds to thousands of genes in each of the gene modules correlated with autistic traits, a comparison analysis of the upstream regulators of these genes reveals that relatively few genes can potentially regulate hundreds of trait-associated genes in both simplex and multiplex populations (Table 7). HNF4A, TP53, and ESR1 are also the top three upstream regulators of the overlapping genes in the simplex and multiplex samples (*p*-values of overlap: 2.48E-25, 7.8E-23, and 1.06E-20, respectively).

**Table 6:** Top 25 canonical pathways significantly over-represented among overlapping genes associated with all traits in simplex and multiplex populations.

**Ingenuity canonical pathways**	–log(*p*-value)^*^
Molecular Mechanisms of Cancer	8.31
Cell Cycle: G2/M DNA Damage Checkpoint Regulation	6.85
ATM Signaling	6.64
**Estrogen Receptor Signaling**	6.37
Senescence Pathway	6.23
Hypoxia Signaling in the Cardiovascular System	6.16
Cyclins and Cell Cycle Regulation	6.16
PI3K Signaling in B Lymphocytes	6.08
**p70S6K Signaling**	5.55
Cell Cycle Control of Chromosomal Replication	5.24
**Protein Ubiquitination Pathway**	5.19
**Synaptogenesis Signaling Pathway**	5.03
**Regulation of eIF4 and p70S6K Signaling**	5.03
**Reelin Signaling in Neurons**	4.85
Pyridoxal 5′-phosphate Salvage Pathway	4.8
ErbB4 Signaling	4.68
B Cell Receptor Signaling	4.5
**NGF Signaling**	4.45
**Protein Kinase A Signaling**	4.15
**Opioid Signaling Pathway**	4.12
Sumoylation Pathway	4.11
Systemic Lupus Erythematosus In B Cell Signaling Pathway	4.02
Natural Killer Cell Signaling	4
**Ephrin Receptor Signaling**	4
GM-CSF Signaling	3.93

* Negative logarithm of the Fisher Exact p-value which indicates the probability that the indicated pathway is not over-represented in the respective dataset of genes; a value of 1.3 is equivalent to a p-value of 0.05.

*Boldface font indicates association with ASD*.

**Table 7:** Comparison of the most significant over-represented canonical pathways and upstream regulators associated with gene modules correlated with all traits in simplex and multiplex ASD populations.

**Canonical pathways**	**Simplex**	**Multiplex**
**(All traits combined)**	**–log (*****p***-value)^*^	
Molecular Mechanisms of Cancer	11.32	16.83
**Regulation of eIF4 and p70S6K Signaling**	11.02	11.41
**Estrogen Receptor Signaling**	6.63	13.43
ATM Signaling	13.32	6.67
Senescence Pathway	10.76	8.97
**Protein Ubiquitination Pathway**	8.80	9.29
**EIF2 Signaling**	9.55	8.48
**p70S6K Signaling**	6.92	11.01
B Cell Receptor Signaling	5.64	11.40
Hepatic Fibrosis Signaling Pathway	0.97	15.96
**mTOR Signaling**	9.11	7.56
**Protein Kinase A Signaling**	4.03	12.29
Huntington's Disease Signaling	6.58	9.18
**Synaptogenesis Signaling Pathway**	5.81	9.86
PI3K Signaling in B Lymphocytes	6.02	9.59
Thrombin Signaling	3.11	11.79
Hereditary Breast Cancer Signaling	6.54	8.35
**Axonal Guidance Signaling**	2.31	12.48
**Ephrin Receptor Signaling**	7.40	7.35
Hypoxia Signaling in the Cardiovascular System	9.70	4.74
**Upstream Regulators**	**Simplex**	**Multiplex**
**(All traits combined)**	***p***-value of overlap^*^	**(# target genes)**
HNF4A	1.86E-59 (728)	3.19E-46 (821)
TP53	7.66E-25 (559)	3.85E-49 (761)
ESR1	9.81E-16 (396)	5.86E-54 (606)
MYC	1.57E-14 (353)	9.34E-30 (485)
beta-estradiol	7.42E-08 (525)	2.30E-35 (791)

* Negative logarithm of the Fisher Exact p-value which represents the probability that the pathway is not over-represented among the respective dataset of genes, where a value of 1.3 is equivalent to a p-value of 0.05.

Bold font indicates association with ASD.

* *Indicates the probability that the trait-associated genes are not among those known to be regulated by the indicated upstream regulator*.

## Discussion

### The Advantage of Phenotypic Subtyping in ASD

A primary goal of this study was to test the hypothesis that reducing the heterogeneity of ASD by subtyping cases according to clinical severity across a broad spectrum of autistic behaviors and traits will improve upon the ability to identify DEGs together with their associated pathways and biological functions in each of the subgroups relative to the combined set of cases. In this study, we apply multivariate cluster analyses of ADI-R scores to divide individuals with ASD from simplex families included in the SSC into three clinically distinct subgroups and perform gene expression profiling of each subgroup using existing transcriptomic data from a published study. In contrast to our study, the Luo et al. study (22) analyzed the expression outliers in individual pairs of case-control siblings and correlated them with CNVs from the same individuals. Here, we are interested in using the transcriptomic data to identify DEGs between probands and their respective unaffected siblings in each of the phenotypic subgroups in order to detect biological differences between cases and controls that associate with the differences in the severity of specific clinical symptoms. While we have previously demonstrated that this subtyping strategy improved upon the biological information obtained on ASD subgroups derived from multiplex families (7), this approach has never been used to analyze transcriptomic data from simplex families. Our results show that: (1) significant DEGs can successfully separate cases from controls (Figure 3) in each of the three subtypes derived from the ADI-R cluster analyses, and (2) the DEGs in each subgroup are differentially enriched in autism-related canonical pathways, diseases and biological functions (Tables 1, 2). Notably, despite the fact that the Language subgroup is comprised of the fewest number of samples (seven pairs), transcriptomic and pathway prediction analyses show that DEGs from this group are the most enriched in autism genes (as reflected by a hypergeometric distribution *q*-value of 0.001 for enrichment in SFARI genes), pathways, and functions relevant to ASD. Among the significantly enriched ASD-relevant pathways associated with the Language phenotype are those involved in axonal guidance, actin cytoskeleton signaling, synaptogenesis, GABA receptor, and neuregulin signaling (Table 1). By contrast, DEGs from the Intermediate and Mild subgroups are enriched in fewer pathways related to ASD. Interestingly, when all 73 cases were combined for transcriptomic and functional analyses, none of the canonical pathways over-represented within the resulting dataset of DEGs were specifically associated with ASD (Supplementary Table 7). Similarly, with respect to neurological diseases, the Language subtype exhibits the largest number of functions and comorbid disorders associated with ASD, including movement disorders and cognitive impairment. By contrast, the Intermediate and Mild subtypes are associated with sensory disorders and epilepsy, respectively (Table 2). These findings thus demonstrate that the reduction in heterogeneity of ASD cases within the simplex population helps to reveal significant but different biological processes and disorders associated with each subtype of autism based on gene expression profiles. We have recently demonstrated that application of this subtyping method to DNA methylation data from probands in simplex families similarly enhances the ability to identify subtype-associated genes in differentially methylated regions (DMRs) in the same three phenotypic subgroups represented in this study (34). Moreover, 1.6 times more DMR-associated genes are detected among the three subgroups in comparison to those detected when all cases are combined into a single case group, again demonstrating the value of heterogeneity reduction and phenotype definition for genome-wide omics analyses. Together, these gene expression and methylation studies reveal dysregulated ASD subtype-dependent genes, pathways, biological functions, and gene regulatory mechanisms that may aid in the design of therapeutic strategies for each phenotypic subgroup.

### Biological Associations With Autistic Traits in Simplex Families

While WGCNA has been primarily used to identify gene modules that correlate with a specific disease state (e.g., autism, Alzheimer's disease, bipolar disorder, renal and pancreatic cancer), in this study, we use WGCNA to identify gene modules that may relate to specific traits of ASD. These traits included deficits in verbal ability (spoken language), non-verbal communication, play skills, social interaction, insistence on sameness/rituals, and manifestation of savant skills. As shown in Figure 4, seven modules were significantly correlated with at least one of three traits (verbal, non-verbal, and social), with the non-verbal trait associated with the largest number of ASD-related pathways. Interestingly, all seven modules were also identified in autism brain co-expression networks (16), suggesting relevance of our results to neurological processes impacted in the autistic brain. Separate network prediction analyses of genes in all of the modules correlated with each trait revealed both overlapping and unique canonical pathways associated with each trait, as summarized in Figure 5. Among the shared pathways are those involving regulation of eIF4 and p70S6K signaling as well as ERK/MAPK, mTOR, and androgen signaling, all of which have been implicated in ASD (7, 35–40).

### Biological Associations With Autistic Traits in Individuals From Multiplex Families

Although we have previously reported on differential gene expression in three subtypes of ASD from multiplex families (7), we were interested in applying WGCNA to study the correlation of autistic traits in this population with gene networks in order to compare the results with those from the simplex population, which was the main focus of this study. Figure 6 shows that many more gene modules can be correlated with autistic traits in individuals from multiplex families than from simplex families. In addition, each of five autistic traits (verbal, non-verbal, play, insistence on sameness, and savant skills) could be correlated with at least one gene module. In particular, the verbal trait is associated with 13 modules, two each for non-verbal and play skills, three for insistence on sameness, and seven for savant skills. The majority of these modules, with the exception of plum1 and darkolivegreen, were also found among co-expression networks in the autistic brain (16). In contrast to the results from the simplex population, the verbal trait is associated with the largest number of pathways implicated in ASD while the non-verbal trait is associated with relatively few ASD-relevant pathways (Table 4). Interestingly, the Language subgroup in the multiplex population accounts for 34.1% of the 1,954 individuals for whom ADI-R scores were available. In the simplex population, the Language subgroup makes up only 11.1% of the 1,900 probands for whom ADI-R scoresheets were obtained. It is also notable that savant skills show inverse correlation with other autistic traits sharing the same modules, suggesting that the presence of savant skills may counteract at least in part the severity of some autistic traits. In other words, the direction of changes in gene expression associated with the presence of savant skills may be opposite to that which is associated with other autistic traits. By contrast, we did not find any modules that correlated with savant skills in probands from the simplex population. It is noted that one subgroup of individuals with ASD (called the “Savant” subgroup) in the multiplex population that was included in our previous gene expression profiling analyses exhibited a higher frequency of savant skills than individuals in the other three subgroups (4, 7). The Savant subgroup was not distinguished within the simplex population, which instead had a higher percentage of individuals in the Mild subgroup (52.5%). Thus, the larger number of trait scores related to savant skills in the multiplex population may account in part for the ability to identify gene modules correlated with this trait.

### Similarities and Differences Associated With ASD Traits in Simplex and Multiplex Populations

The trait-associated genes in both simplex and multiplex populations are enriched in autism-risk genes from the SFARI Gene database, but the significance of the enrichment is greater for the multiplex population by ~4 orders of magnitude. Likewise, the number of trait-associated modules (excluding gray) are considerably greater for the multiplex population (18 vs. 7 modules). These results suggest that there may be a greater burden with respect to gene dysregulation and network disruption in individuals with ASD from multiplex families. Despite these differences, there are shared canonical pathways that appear to underlie ASD traits in both populations albeit to different extents, as shown in Table 7. Interestingly, many of the trait-associated genes in both populations can be regulated by a handful of potent upstream regulators, in particular, HNF4A, TP53, ESR1, MYC, and estradiol. The tumor suppressor gene, *TP53*, and the proto-oncogene *MYC* are both regulators of cell cycle progression, growth, and apoptosis, processes that are notably deregulated in ASD (36, 41). The estrogen receptor 1, ESR1, as well as its ligand estradiol, are critically important to brain development and sexual differentiation (42, 43), and have been implicated in a number of studies on ASD (44, 45). Hepatocyte nuclear factor 4, HNF4A, is a transcription factor known to play a major role in liver and gastrointestinal diseases (46). While HNF4A has never before been associated specifically with ASD, it has been reported to be involved in Parkinson's disease (47) and major depressive disorder (48) as well as in regulation of circadian rhythm (49). Notably, circadian rhythm and sleep disorders have previously been associated with ASD by clinical, genetics, and transcriptomic analyses (7, 26, 50–52). This study shows that HNF4A may also disrupt a large number of genes and pathways associated with autistic traits and, in addition, serve as a link to gastrointestinal disorders, such as diarrhea, constipation, inflammatory bowel disease and colitis, which are comorbidities in some individuals with ASD (26, 53–55). While none of these upstream regulators are considered autism risk genes themselves, our findings suggest that therapeutic agents targeted toward these master regulators may have a significant impact on many downstream pathways associated with the autistic phenotype.

### Limitations and Future Considerations

An obvious limitation of this study is that our results are based on transcriptomic analyses of lymphoblastoid cell lines. It has been argued that peripheral tissues are not the ideal experimental model to understand brain development despite the fact that many neurological functions and signaling pathways relevant to autism have been identified in LCL and other peripheral tissues, such as whole blood, lymphocytes, and natural killer cells (7, 56–62). In addition, the extensive overlap of trait-associated gene modules (18 out of 20) from this study with those resulting from transcriptomic analysis of the autistic brain (16) suggests that LCL are a useful surrogate model for investigations of the pathobiology underlying ASD. Another limitation is that sample sizes are small, especially for the Language subtype, even though the largest number of DEGs and more ASD associated pathways and biological functions were identified for this subgroup compared to the other two subgroups that have 3.7–5.8 times more cases. Moreover, only male probands were used for the WGCNA analyses of autistic traits. Future studies should include additional samples in each of the phenotypic subgroups to confirm the DEGs and pathways reported here as well as more females to allow a separate WGCNA analysis of autistic traits in females.

## Conclusions

This study shows that heterogeneity reduction by phenotypic subtyping of individuals with ASD enhances the ability to identify more differences in autism-relevant gene expression, pathways, and functions between probands and siblings from simplex families in comparison to those obtained with a combined case group. These findings thus replicate those of our earlier study that applied this subtyping approach to transcriptomic analyses of the multiplex ASD population. While we used multivariate cluster analyses of ADI-R severity scores for the subtyping, we anticipate that other approaches to reduce heterogeneity among individuals with ASD (e.g., by comorbidities, immune status, physical abnormalities (like head size), or functional MRI profiles) may also improve the ability to detect biological differences at the genome-wide level.

To our knowledge, this is the first study to use WGCNA to analyze gene networks in the context of a continuous severity spectrum of autistic traits rather than with a dichotomous diagnosis of ASD. Perhaps the most important findings from this study are the associations of specific ASD traits with expressed gene networks and embedded pathways that may serve as useful targets for precision medicine in autism spectrum disorders.

## Data Availability Statement

Publicly available datasets were analyzed in this study. This data can be found here: Gene Expression Omnibus (GEO); Accession numbers GSE37772 and GSE15402.

## Ethics Statement

The studies involving human participants were reviewed and approved by GWU Institutional Review Board, Office for Human Research. Written informed consent from the participants' legal guardian/next of kin was not required to participate in this study in accordance with the national legislation and the institutional requirements.

## Author Contributions

VH conceived of and designed the study, performed subgroup phenotyping, pathway, functional and hypergeometric analyses, and wrote the manuscript. CB was responsible for gene expression and WGCNA analyses as part of her Master's thesis research and contributed to manuscript preparation. All authors contributed to the article and approved the submitted version.

## Conflict of Interest

The authors declare that the research was conducted in the absence of any commercial or financial relationships that could be construed as a potential conflict of interest.

## Supplementary Material

Supplementary Table 1 Demographic information on the individuals from the SSC population included in this study.

Supplementary Table 2 Clinical autistic traits classification and included trait-associated ADI-R items.

Supplementary Table 3 Differentially expressed genes in the Language subtype of ASD.

Supplementary Table 4 Differentially expressed genes in the Intermediate subtype of ASD.

Supplementary Table 5 Differentially expressed genes in the Mild subtype of ASD.

Supplementary Table 6 Differentially expressed genes in the combined case group.

Supplementary Table 7 All significant over-represented canonical pathways associated with DEGs in the subtypes of ASD and the combined case group.

Supplementary Table 8 All significant over-represented canonical pathways associated with module genes corresponding to verbal, non-verbal, social, and all (combined) traits in the simplex population.

Supplementary Table 9 All significant over-represented canonical pathways associated with module genes corresponding to verbal, non-verbal, play, insistence on sameness, savant, and all (combined) traits in the multiplex population.
