# Supplementary material for: Phenotypic Subtyping and Re-analyses of Existing Transcriptomic Data from Autistic Probands in Simplex Families Reveal Differentially Expressed and ASD Trait-Associated Genes
Source: Front Neurol. 2020 Nov 12;11:578972. doi: 10.3389/fneur.2020.578972 (PMC7689346; doi:10.3389/fneur.2020.578972)
Supplement: Supplementary Table 1 — Demographic information on the individuals from the SSC population included in this study. [file Table_1.docx]

Table S1: Demographic information on individuals from the SSC population in this study

|  | **Probands** | **Siblings** |
| --- | --- | --- |
| Number of samples | 74 | 74 |
| Gender (male:female) | 63:11 | 63:11 |
| Age (years) | 10.40 ± 3.81 | 10.32 ± 3.83 |
| Subtypes (# samples) | Language (7)  Intermediate (26)  Mild (41) | Language (7)  Intermediate: 26  Mild (41) |
