# Supplementary material for: Phenotypic Subtyping and Re-analyses of Existing Transcriptomic Data from Autistic Probands in Simplex Families Reveal Differentially Expressed and ASD Trait-Associated Genes
Source: Front Neurol. 2020 Nov 12;11:578972. doi: 10.3389/fneur.2020.578972 (PMC7689346; doi:10.3389/fneur.2020.578972)
Supplement: Supplementary Table 2 — Clinical autistic traits classification and included trait-associated ADI-R items. [file Table_2.docx]

Table S2: Clinical autistic traits classification and included trait-associated ADI-R items

| Communication and language impairment | | Quality of social interaction | | Repetitive, restricted and stereotyped interests and behaviors | |
| --- | --- | --- | --- | --- | --- |
| Verbal communication (n= 20 items) | Nonverbal communication (n = 15 items) | Play skills  (n = 14 items) | Social development (n = 21 items) | Insistence on Sameness-rituals  (n = 16 items) | Savant skills  (n = 12 items) |
| CARTIC | COMPSL | CPLAY | GAZE5 | CUNPROC | CVISSP |
| ARTIC5 | COMSL5 | PLAY5 | CSSMILE | EUNPROC | EVISSP |
| CSTEREO | CUSEBOD | CPEERPL | SSMILE5 | CCIRINT | CMEM |
| ESTEREO | EUSEBOD | PEERPL5 | CSHOW | ECIRINT | EMEM |
| CCHAT | CPOINT | CSOPLAY | SHOW5 | CUSEOBJ | CMUSIC |
| CHAT5 | POINT5 | SOPLAY5 | COSHARE | EUSEOBJ | EMUSIC |
| CCONVER | CNOD | CINTCH | OSHARE5 | CCRIT | CDRAW |
| CONVER5 | NOD5 | INTCH5 | CSHARE | ECRIT | EDRAW |
| CINAPPQ | CHSHAKE | CRESPCH | SHARE5 | CUNSENS | CREAD |
| EINAPPQ | HSHAKE5 | RESPCH5 | COCOMF | EUNSENS | EREAD |
| CPRON | CINSGES | CGRPLAY | OCOMF5 | CCHANGE | CCOMPU |
| EPRON | INSGES5 | GRPLAY5 | CQUALOV | ECHANGE | ECOMPU |
| CNEOID | AVOICE5 | CFRIEND | QUALOV5 | CRESIS |  |
| ENEOID | CIMIT | FREND15 | CRFACEX | ERESIS |  |
| CVERRIT | IMIT5 |  | RFACEX5 | CUATT |  |
| EVERRIT |  |  | CINAPFE | EUATT |  |
| CINR |  |  | INAPFE5 |  |  |
| EINR |  |  | CQRESP |  |  |
| CSPEECH |  |  | QRESP5 |  |  |
| SPEECH5 |  |  | CINITIA |  |  |
|  |  |  | INITIA5 |  |  |
